# Modulation of Morphogenesis by Egfr during Dorsal Closure in *Drosophila*

**DOI:** 10.1371/journal.pone.0060180

**Published:** 2013-04-08

**Authors:** Weiping Shen, Xi Chen, Olga Cormier, David Chung-Pei Cheng, Bruce Reed, Nicholas Harden

**Affiliations:** 1 Department of Molecular Biology and Biochemistry, Simon Fraser University, Burnaby, British Columbia, Canada; 2 Department of Biology, University of Waterloo, Waterloo, Ontario, Canada; University of Massachusetts Medical School, United States of America

## Abstract

During *Drosophila* embryogenesis the process of dorsal closure (DC) results in continuity of the embryonic epidermis, and DC is well recognized as a model system for the analysis of epithelial morphogenesis as well as wound healing. During DC the flanking lateral epidermal sheets stretch, align, and fuse along the dorsal midline, thereby sealing a hole in the epidermis occupied by an extra-embryonic tissue known as the amnioserosa (AS). Successful DC requires the regulation of cell shape change via actomyosin contractility in both the epidermis and the AS, and this involves bidirectional communication between these two tissues. We previously demonstrated that transcriptional regulation of myosin from the *zipper* (*zip*) locus in both the epidermis and the AS involves the expression of Ack family tyrosine kinases in the AS in conjunction with Dpp secreted from the epidermis. A major function of Ack in other species, however, involves the negative regulation of Egfr. We have, therefore, asked what role Egfr might play in the regulation of DC. Our studies demonstrate that Egfr is required to negatively regulate epidermal expression of *dpp* during DC. Interestingly, we also find that Egfr signaling in the AS is required to repress *zip* expression in both the AS and the epidermis, and this may be generally restrictive to the progression of morphogenesis in these tissues. Consistent with this theme of restricting morphogenesis, it has previously been shown that programmed cell death of the AS is essential for proper DC, and we show that Egfr signaling also functions to inhibit or delay AS programmed cell death. Finally, we present evidence that Ack regulates *zip* expression by promoting the endocytosis of Egfr in the AS. We propose that the general role of Egfr signaling during DC is that of a braking mechanism on the overall progression of DC.

## Introduction

Dorsal closure (DC) is a developmental event occurring in the *Drosophila* embryo between stages 11 and 16, commencing immediately after germband retraction (reviewed in [1]). Upon the completion of germband retraction, a large dorsal opening is evident in the epidermis. The amnioserosa (AS), an extra-embryonic tissue composed of a single layer of large flat epithelial cells, spans the opening. The two opposing lateral epidermal flanks elongate in the dorsal-ventral (D-V) axis and move dorsal ward to seal the dorsal hole. The dorsal-most epidermal (DME) cells from one side of the embryo meet their counterpart DME cells at the dorsal midline. The epidermal sealing process occurs in a zipper-like manner, progressing simultaneously from both the anterior and posterior ends of the dorsal opening and finishing at the center of the dorsal midline. During DC, the AS contracts and its cells become more cuboidal in shape; the AS also actively extrudes approximately 10% of its cells with the effect of increasing the rate of DC [2]–[4]. Upon the completion of DC, the entire AS degenerates by programmed cell death [2].

The DME cells experience a range of morphogenetic events during DC. These include elongation in the D-V axis, formation of actin-based membrane extensions, and adhesion with their partners from the other side of the embryo. DC is a popular model system to study cell shape change in epithelial morphogenesis and multiple signaling proteins have been characterized in this context. In addition to signaling pathways and proteins, there are a number of mechanical forces driving DC [4](reviewed in [5]). These include a supracellular actomyosin cable that is assembled at the leading edge (LE) of the DME cells to form a contractile “purse string”. This contractile apparatus constricts the DME cells in the anterior-posterior axis and thus contributes to their stretching in the D–V axis and movement towards the dorsal midline. Actin-based filopodia and lamellipodia also project from the leading edge of the DME cells, and these are thought to contribute to the alignment and adhesion of opposing DME cells as DC concludes [6], [7]. Finally, as was demonstrated by elegant laser micro-dissection experiments, AS constriction not only removes this tissue as an impediment to movement of the epidermis, but also pulls the DME cells dorsal ward [4].

Among the numerous signaling proteins known to regulate DC is Dpp, a member of the transforming growth factor-β superfamily of cytokines. Dpp expression in DME cells is required for morphogenesis of both the amnioserosa and the epidermis during DC. This requirement for Dpp expression in the DME cells is associated, at least in part, with the regulation of the expression of *zipper* (*zip*), which encodes non-muscle myosin II heavy chain [8]–[26]. We previously demonstrated that two members of the Ack family of nonreceptor tyrosine kinases, Ack and PR2, co-operate with Dpp to regulate myosin levels in the AS and epidermis during DC. This work led us to propose the existence of a diffusible signal that is generated by the AS and is regulated by Ack and PR2 in conjunction with Dpp [26]. The *zip* product is required for cell shape change in both the AS and the epidermis; *zip* expression, which is regulated by the Ack/PR2/Dpp signaling network, may ultimately coordinate the overall progression of DC [27].

There is considerable evidence that a major function of Ack is the negative regulation of Egfr, and this is thought to occur through the regulation of Egfr by endocytosis and/or ubiquitination [28]–[32]. Egfr may, therefore, play a key role in the Ack/PR2/Dpp regulatory pathway during DC. The Egfr pathway is used repeatedly throughout *Drosophila* development and appears to regulate a myriad of processes including cell proliferation, cell differentiation, apoptosis, cell motility and adhesion (reviewed in [33], [34]). While it has long been recognized that Egfr has multiple roles in regulating morphogenesis, including germband retraction, its role in DC has not been specifically addressed [35], [36].

The results presented here demonstrate that Egfr is required in both the AS and epidermis for proper DC. In addition, we demonstrate that the function of Egfr in the AS involves the transcriptional repression of *zip* in both the AS and the DME cells, and we suggest that this repression involves the regulation of the same diffusible signal previously proposed to be regulated through Ack and PR2. Consistent with this, we present results suggesting that Ack negatively regulates Egfr in the AS by targeting it for endocytosis. Similar to Ack, we suggest that Egfr regulates *zip* expression in parallel to Dpp signaling, but we find that Egfr also has a strong negative effect on epidermal Dpp transcription. Finally, we confirm that Egfr signaling has an additional role in the AS as an inhibitor of apoptosis. The various roles identified for Egfr signaling during DC are consistent with negative regulation of morphogenesis, and we propose that Egfr acts as a brake to adjust the rate of closure in response to endocytic regulation.

## Results

### Egfr is Required for Normal DC

**Figure 1:**
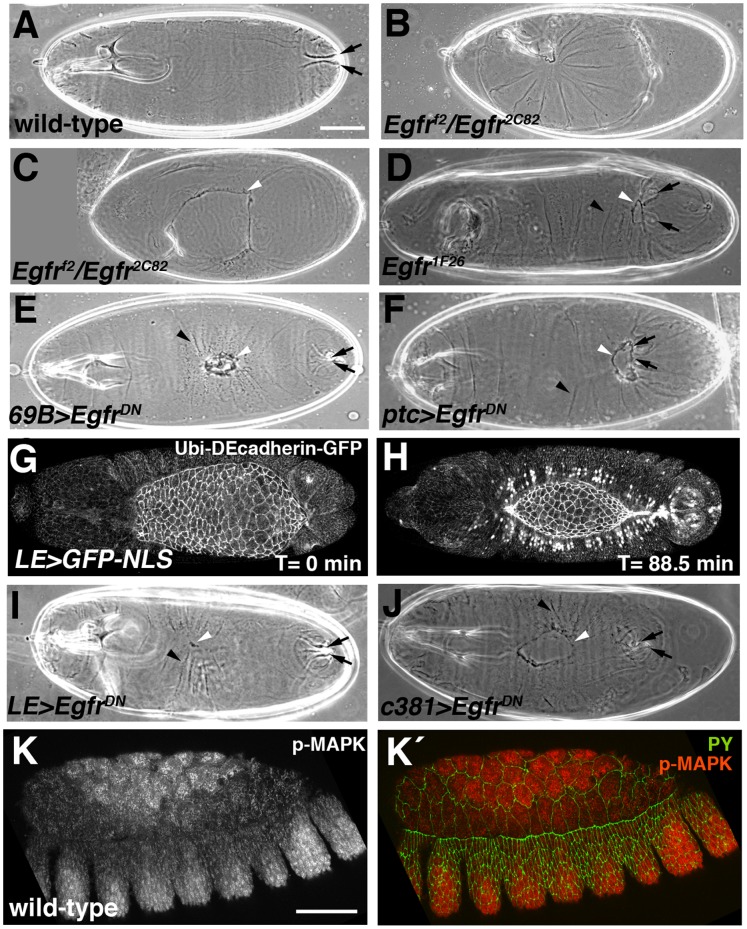
Embryos with either global or local loss of Egfr signaling have defects in epithelial morphogenesis. (A–F, I, J) Cuticle preparations. Black arrows, posterior spiracles; black arrowheads, puckers in cuticle; white arrowheads, dorsal hole or scab. (G, H, K, K´) Confocal micrographs of embryos during dorsal closure (DC). (A) Dorsolateral view of wild-type embryo showing intact dorsal surface. (B) *Egfr^f2^*/*Egfr^2C82^* embryo, selected by absence of GFP balancer chromosome, showing “curled up” phenotype. (C) *Egfr^f2^*/*Egfr^2C82^* embryo showing large dorsal hole. (D) Embryo from temperature-sensitive *Egfr^1F26^* stock that had been allowed to develop at 18°C before shifting to restrictive temperature of 29°C at about stage 10/11. Embryo has a bowed appearance characterized by pulling in of tail (marked by posterior spiracles) and puckering of cuticle. There is a small dorsal hole in the cuticle. (E) Embryo in which *Egfr^DN^* had been expressed in the epidermis using the *69B-Gal4* driver showing dorsal scab and mild bowing. (F) Embryo in which *Egfr^DN^* had been expressed in the epidermis using the *ptc-Gal4* driver showing bowed appearance and dorsal hole, similar to the embryo in panel D. (G, H) Still images from Movie S1 showing restricted expression pattern of *LE-Gal4* driver, revealed using a *UAS-GFP-NLS* reporter. Cell outlines were revealed through expression of a *Ubi-DEcadherin-GFP* transgene. (G) *LE-Gal4* is not expressed at beginning of DC. (H) Midway through DC, GFP-NLS is expressed in the first two rows of cells flanking the amnioserosa (AS), visualized as GFP signal in nuclei. (I) Embryo in which *Egfr^DN^* had been expressed using the *LE-Gal4* driver showing mild bowing and dorsal scab. (J) Embryo in which *Egfr^DN^* had been expressed in the AS using the *Gal4^c381^* driver showing small dorsal hole. (K) Anti-phospho-MAPK staining of a wild-type embryo showing strong immunoreactivity in the center of the amnioserosa and lateral epidermis but little staining in dorsal epidermis and cells at periphery of the AS. (K´) Same embryo as in K with phospho-MAPK in red and cell outlines revealed with anti-phosphotyrosine (PY, green). Scale bars: 50 µm (A–J)(K, K´).

To address the potential function of Egfr in DC we chose three alleles previously shown to disrupt embryogenesis: *Egfr^f2^*, a severe loss-of-function allele, *Egfr^2C82^*, a moderate loss-of-function allele, and *Egfr^1F26^*, a conditional allele [35]–[37]. Two previously uncharacterized embryonic lethal alleles, *Egfr^1a15^* and *Egfr^H25^*, were also used in the course of this study [38]. Phenotypic analysis of the latter two alleles indicated that *Egfr^1a15^* is a severe loss-of-function allele (equivalent to *Egfr^f2^*) and that *Egfr^H25^*is also a strong loss-of-function allele, but is slightly less severe than either *Egfr^f2^*or *Egfr^1a15^* (data not shown). Cuticle preparations of embryos heteroallelic for *Egfr^f2^* and *Egfr^2C82^* showed severe defects in morphogenesis. Greater than 95% of embryos exhibited the previously described “faint little ball” phenotype [36], [37], being “curled” up, with the posterior end of the embryo in close proximity to the head, indicating a defect in germband retraction (Fig. 1B). In less severely curled embryos it was possible to observe holes in the dorsal surface that typically extended anteriorly into the head (Fig. 1C). In general, severe *Egfr* mutants were associated with a terminal phenotype that included severe defects in head development as well as a complete failure in germ band retraction – both of which effectively precluded any analysis of DC. We, therefore, sought approaches that would allow us to observe DC defects in individuals with impaired Egfr function. The first of the two approaches involved temperature shift experiments using the conditional allele *Egfr^1F26^* [35]. Embryos collected at the permissive temperature of 18°C were aged for various periods of time before being shifted to the restrictive temperature of 29°C. Embryos transferred to 29°C at any stage prior to stage 10 showed severe cuticle defects similar to those seen with other strong loss-of-function *Egfr* alleles (data not shown). Shifting slightly older (approximately stage 11) *Egfr^1F26^* mutant embryos to 29°C, however, resulted in a less severe mutant phenotype comprising a smaller head hole, distinct from other defects in the dorsal surface, and a less severe defect in germband retraction (Fig. 1D). In addition, these embryos consistently displayed creases or “puckers” in the dorsal surface, which together with the mild germband retraction defect, gave them a bowed appearance. Approximately 52% of *Egfr^1F26^* embryos were scored as having this bowed phenotype in a temperature shift experiment where a 2.5-hour collection of embryos from the *Egfr^1F26^* stock was aged at 18°C for 12 hours and then shifted to 29°C (corresponding to late stage 10/stage 11 at the time of the temperature shift). Of these bowed embryos, half exhibited an additional phenotype of a hole or scab in the dorsal surface (Fig. 1D). Few defects were seen in the dorsal surface of *Egfr^1F26^* embryos when they were shifted to 29°C at stage 12 or later (data not shown).

A second approach for facilitating the analysis of DC defects in Egfr-deficient embryos, which also permitted characterization of the tissue specificity of Egfr function, involved the inducible expression of a dominant negative version of *Egfr*, *Egfr^DN^*. *Egfr^DN^* competes with endogenous Egfr for ligand binding, but lacks the cytoplasmic domain, which contains the tyrosine kinase domain necessary for trans-phosphorylation and receptor activation [39]. As a result, Egfr^DN^ attenuates activation of the Egfr cascade in a cell autonomous manner. We expressed a *UAS-Egfr^DN^* transgene in various spatial patterns, starting with general epidermal expression and epidermal stripes using the *69B-Gal4* and *ptc-Gal4* drivers, respectively [40], [41]. Both patterns of Egfr^DN^ expression resulted in bowed embryos with occasional dorsal holes or scabs, similar to the temperature shift phenotype (Fig. 1E, F). 100% of embryos in which Egfr^DN^ was expressed with *ptc-Gal4* were bowed and 10% had a dorsal hole or scab. The dorsal epidermis plays an important role in DC and we impaired Egfr function in this tissue by expressing Egfr^DN^ using the *LE-Gal4* driver, which is active only during DC primarily within a subset of cells in the first two rows of dorsal epidermal cells flanking the amnioserosa (Fig. 1G, H, and Movie S1) [13]. This resulted in a bowed embryo phenotype in about a quarter of *Egfr^DN^*-expressing embryos (Fig. 1I). Bowed embryos and dorsal holes were also seen when Egfr activity was blocked in the AS using the AS-specific driver *Gal4^c381^* [42](Fig. 1J). We conclude that the normal progression of DC requires Egfr signaling in both the epidermis and the AS.

A major route for signaling by Egfr is the Raf-MAPK pathway, the activation of which can be detected using anti-phospho-MAPK antibodies [43], [44]. We observed strong phospho-MAPK immunoreactivity in the central AS cells of wild-type embryos, but little staining in cells of the AS periphery or in the dorsal epidermis (Fig. 1 K, K’). This result suggests that either Egfr is not using the MAPK pathway in these cells or that the pathway is under tight negative control. Consistent with Egfr signaling generating phospho-MAPK in the AS, the anti-phospho-MAPK immunoreactivity in the AS was absent in embryos in which *Egfr^DN^* was expressed with *Gal4^c381^* (data not shown).

**Figure 2:**
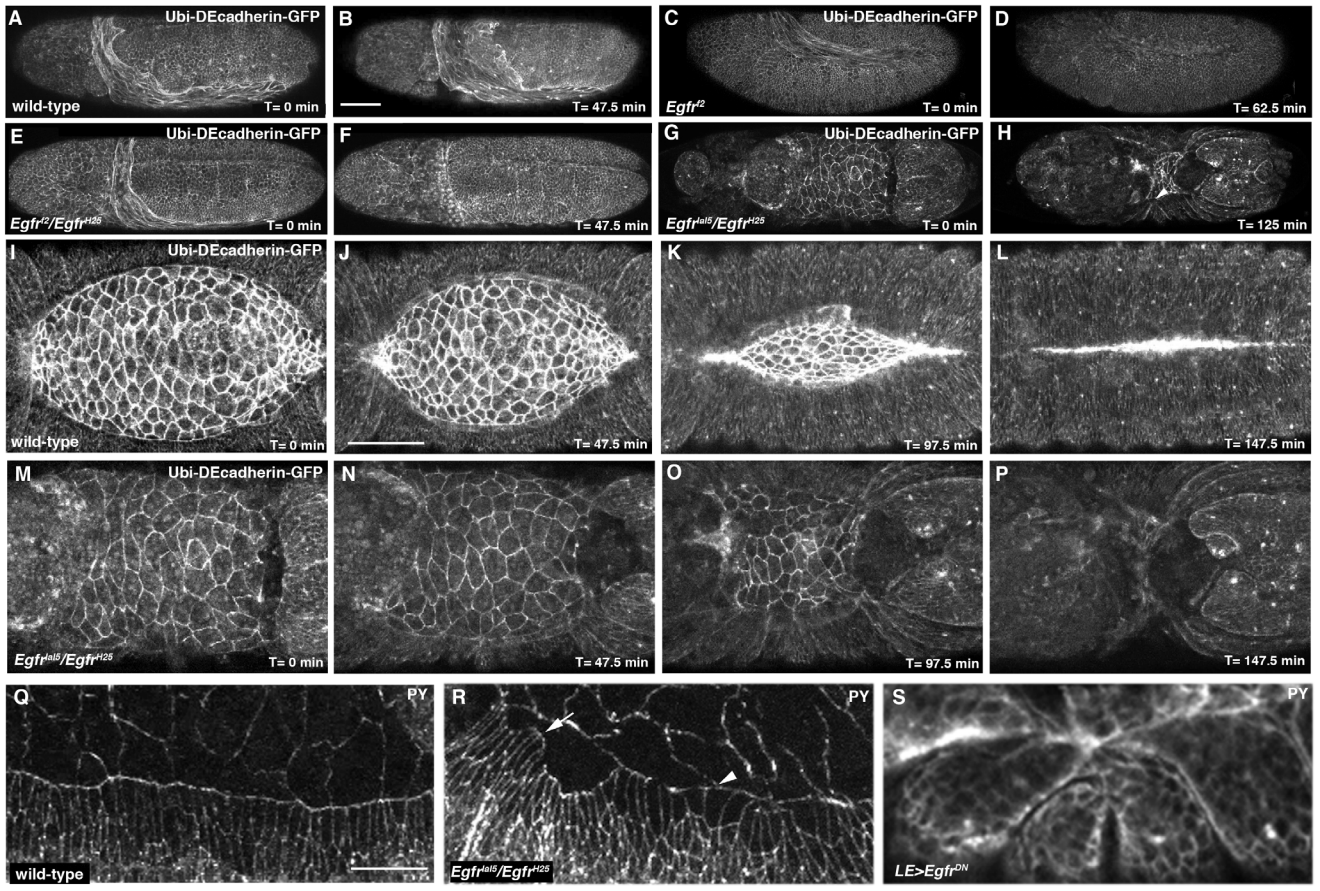
Impairment of Egfr signaling affects morphogenesis of the AS and dorsal epidermis. (A, B) Still images from Movie S2 showing unfolding of the AS as germband retraction proceeds in a *Ubi-DEcadherin-GFP-*expressing embryo. (C, D) Still images from Movie S3 showing delayed germband retraction and disintegration of AS in *Egfr^f2^* mutant embryo expressing *Ubi-DEcadherin-GFP*. (E, F) Still images from Movie S4 showing delayed germband retraction and disintegration of AS in *Egfr^f2^*/*Egfr^H25^* mutant embryo expressing *Ubi-DEcadherin-GFP*. (G, H) Still images from Movie S5 showing bowing of *Egfr^1a15^*/*Egfr^H25^* mutant, *Ubi-DEcadherin-GFP-expressing* embryo. Note bunching of segments (arrowhead in panel H). (I–L) Still images from Movie S­6 showing morphogenesis of the AS in a *Ubi-DEcadherin-GFP-*expressing embryo. (M–P) Close-ups of dorsal surface in still images from Movie S5 showing defective AS morphogenesis in *Egfr* mutant embryo. Note that AS has fewer cells than wild-type and constricts perpendicular to the normal anterior-posterior direction. Note that the posterior end of the embryo moves anteriorly in progression from panel N to panel P as the embryo undergoes bowing. (Q-S) Confocal micrographs of dorsal epidermis of embryos stained with anti-phosphotyrosine. (Q) Wild-type embryo showing uniform shape of DME cells and fairly smooth leading edge. (R) *Egfr^1a15^*/*Egfr^H25^* embryo showing considerable variation in shape of DME cells and jagged leading edge. Arrow marks a cluster of very constricted DME cells and arrowhead a cluster of cuboidal DME cells. (S) Embryo in which *Egfr^DN^* had been expressed using the *LE-Gal4* driver showing bunching of segments. Scale bars: 50 µm (A–H)(I–P)(Q–S).

We extended our analysis of Egfr function in DC by live imaging embryos homozygous or heteroallelic for the alleles *Egfr^f2^* [37], *Egfr^1a15^* and *Egfr^H25^* [38] and carrying a *Ubi-DEcadherin-GFP* transgene to visualize cell outlines [45]. At least four movies were taken for each genotype and very consistent phenotypes were observed. Prior to the initiation of germband retraction, degradation of the AS commenced in *Egfr* mutant embryos, which in the most severe cases led to a complete and dramatic loss of the tissue (Compare Movie S2 to Movies S3 and S4, and Fig. 2A, B to Fig. 2 C, D and Fig. 2 E, F). In some embryos the AS persisted throughout germband retraction and DC, but had noticeably fewer cells than wild-type (Movie S5 and Fig. 2G, H, M–P). In such embryos germband retraction proceeded to a point and then appeared to reverse, with the posterior end of the embryo moving anteriorly. Accompanying this was a bunching of the epidermis characterized by inappropriate adhesion between the dorsal end of non-adjacent segments on the same side of the embryo producing (arrowhead in Fig. 2H). In addition, AS morphogenesis was abnormal, with the tissue constricting perpendicular to the normal anterior-posterior direction (Compare Movie S5 to Movie S6 and Fig. 2I–L to Fig. 2M–P). Finally, heads of *Egfr* mutant embryos exhibited a precipitous loss of epidermal integrity during embryogenesis with the brain becoming exposed during DC and pushing toward the posterior end of the embryo (Fig. 2G, H, Movie S5).

We also assessed morphogenesis in fixed embryos by staining with an antibody against phosphotyrosine to reveal cell outlines. Heteroallelic *Egfr* mutant embryos showed highly variable cell shape change of the DME cells compared to wild-type, suggestive of misregulated actomyosin contractility (Fig. 2Q, R). We suspect that this uneven contractility in the dorsal epidermis underlies the bunching of the segments in *Egfr* mutant embryos, and consistent with this expression of *Egfr^DN^* with *LE-Gal4* led to segmental bunching (Fig. 2S).

### Egfr Negatively Regulates *dpp* Expression in the Epidermis During DC

**Figure 3:**
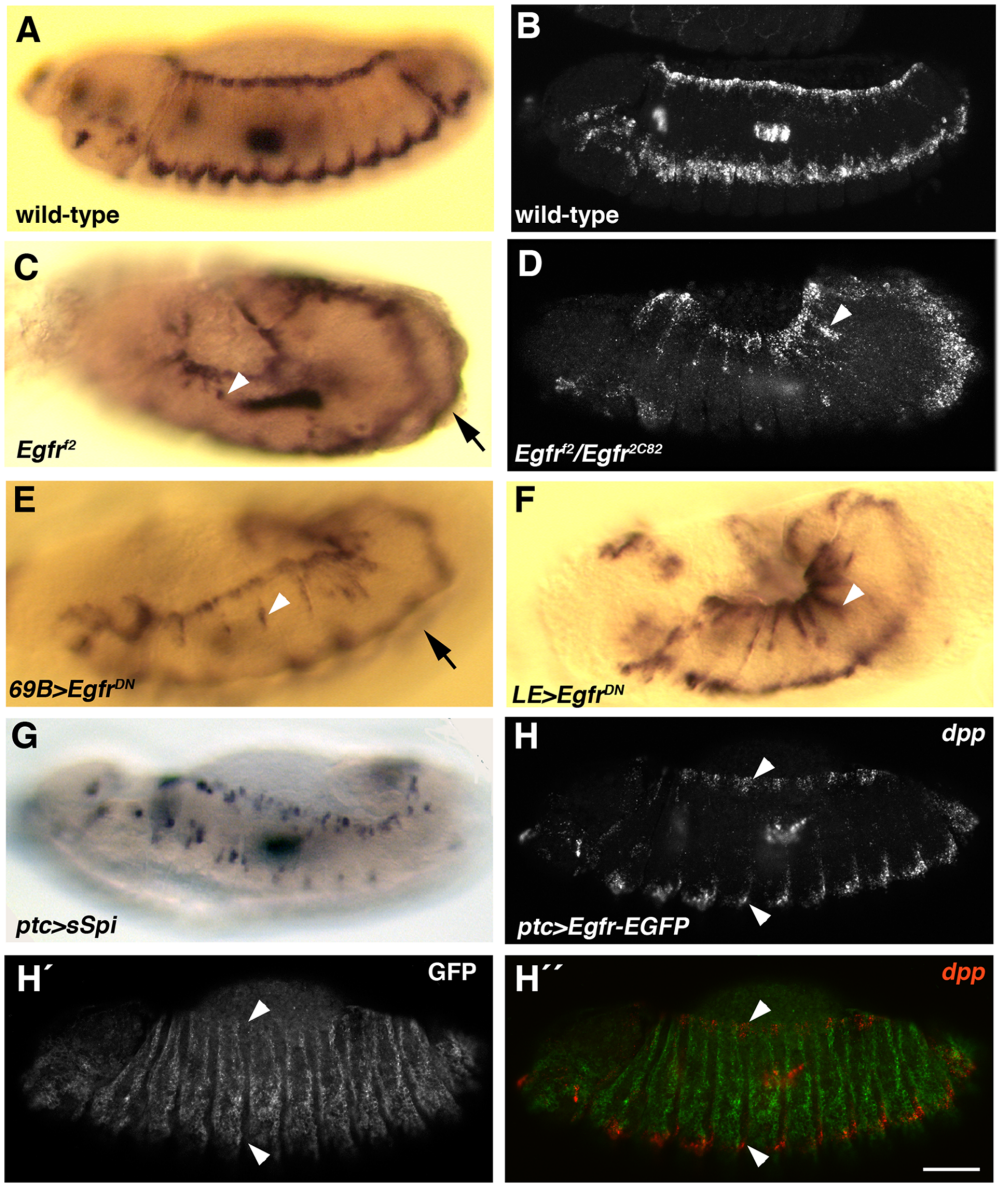
dpp transcription is repressed by Egfr signaling during DC. Panels A, C, E and F are digoxigenin *in situ* hybridizations and panels B, D and H–H´ ´ are FISH, with all embryos at beginning of DC. (A, B) Wild-type embryos showing horizontal dorsal and ventrolateral stripes of *dpp* expression. The dorsal stripe is *dpp* expression in the DME cells. (C, D) *Egfr^f2^* embryo (C) and *Egfr^f2^*/*Egfr^2C82^* embryo (D) showing ectopic *dpp* expression ventral to the DME cells (arrowheads). Arrow in (C) shows ventrolateral stripe visible on other side of embryo due to decreased distance between stripes compared to wild-type. (E) Embryo in which *Egfr^DN^* had been expressed in the epidermis using the *69B-Gal4* driver showing ectopic *dpp* expression (arrowhead). Arrow shows ventrolateral stripe visible on other side of embryo. (F) Embryo in which *Egfr^DN^* had been expressed using the *LE-Gal4* driver showing elevated *dpp* expression in the dorsal epidermis (arrowhead). (G, H–H´ ´) Increasing EGFR signaling by expression of sSpi (G) or Egfr-EGFP (H–H´ ´) in vertical stripes using the *ptc-Gal4* driver causes breaks in the dorsal and ventrolateral *dpp* stripes. Anti-GFP staining (H´, H´ ´) reveals the expression pattern of Egfr-EGFP. Note that remnants of *dpp* expression (arrowheads in H–H´ ´) are seen where Egfr-EGFP was not expressed. Scale bar: 50 µm.

The bowed embryo phenotype associated with reductions in Egfr function is similar to the cuticle phenotype of embryos in which Dpp is ectopically expressed in the dorsal epidermis [17], [46]–[48]. Furthermore, crosstalk between Egfr and Dpp/TGFβ signaling in the form of antagonistic or cooperative interactions has been reported for a number of developmental events [49]–[82]. We, therefore, next examined *dpp* expression in embryos having altered Egfr function. We confirmed a previously published observation that ectopic *dpp* expression extends ventrally along the segmental grooves of *Egfr* mutant embryos [35], and saw a similar pattern of *dpp* expression in embryos in which Egfr signaling was attenuated in the epidermis via *UAS-Egfr^DN^* expression using *69B-Gal4* or *LE-Gal4* drivers (Fig. 3C–F, arrowheads). The phenocopy of *Egfr* loss-of-function mutants by *UAS-Egfr^DN^* expression was underscored by the fact that widespread epidermal expression of this transgene resulted in a reduction in the separation, from one side of the embryo to the other, between the ventral stripes of *dpp* expression as previously reported for *Egfr* and *D-raf* mutant embryos [35], [83]. This decrease in separation allowed the ventral stripe on the other side of the embryo to be seen in a lateral view (arrows in Fig. 3C, E). Given the many studies indicating communication between the AS and epidermis during DC [10], [13], [25], [26], [84]–[89] and the requirement for Egfr in the AS during DC, we looked at *dpp* expression in embryos in which *Egfr^DN^* had been expressed in the AS using *Gal4^c381^*, but found no effect (data not shown).

To examine the effects of excessive Egfr signaling on *dpp* expression we used two transgenes, *UAS-sSpi* and *UAS-Egfr-EGFP. UAS-sSpi* encodes a secreted, active version of the Egfr ligand, Spitz, which can directly bind to Egfr to activate the Egfr pathway, whereas *UAS-Egfr-EGFP* encodes a biologically active Egfr tagged with enhanced green fluorescent protein (EGFP) [90], [91]. These transgenes were expressed in stripes in the embryo using *ptc-Gal4* and effects on *dpp* expression assessed by in situ hybridization. For both transgenes, the *dpp* expression stripes in the dorsal and ventrolateral epidermis became fragmented (Fig. 3G, H). Staining with anti-GFP antibodies revealed that remaining patches of *dpp* expression were in areas where Egfr-EGFP had not been expressed (Fig. 3H ´, H´ ´). We conclude from our loss- and gain-of-function studies that Egfr signaling negatively regulates *dpp* expression in the epidermis during DC.

### Egfr Negatively Regulates *zip* Expression in the Epidermis and AS During DC

**Figure 4:**
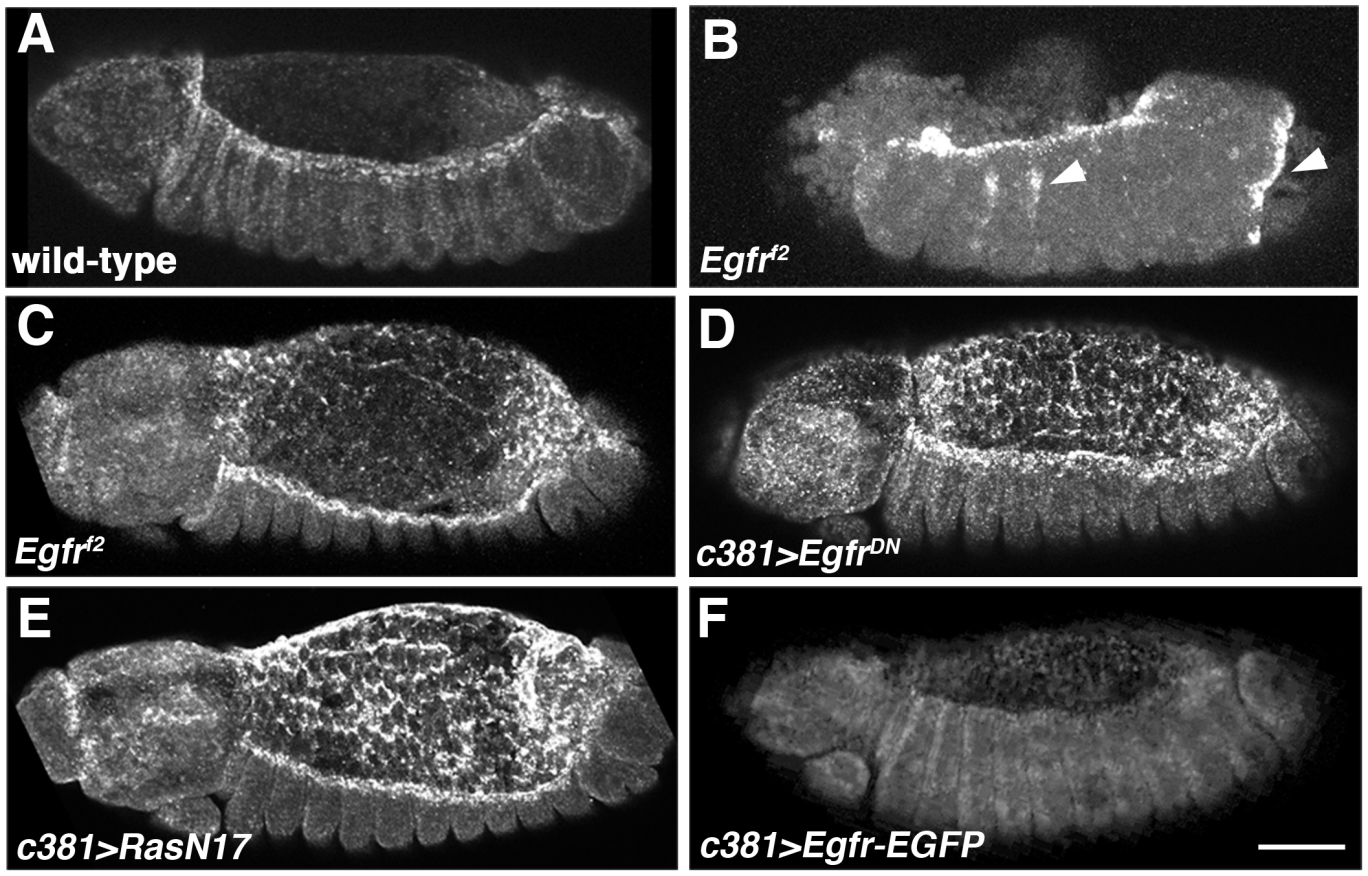
zip transcription is repressed by Egfr signaling during DC. *zip* FISH on embryos at beginning of DC. (A) Wild-type embryo showing high levels of *zip* transcription in DME cells and absence of *zip* expression in the AS. Prior to completion of germband retraction there are high levels of *zip* in the AS of wild-type embryos (see Fig. 6A). (B) *Egfr^f2^* embryo showing intense *zip* signal in DME cells and ectopic *zip* expression (arrowheads). (C) Mildly affected *Egfr^f2^* embryo showing modest retention of *zip* in AS. (D, E) Embryos in which Egfr signaling had been impaired in the AS by expression of either *Egfr^DN^* (D) or *RasN17* (E) showing significant retention of *zip* in AS, modest elevation of *zip* expression in the DME cells and ectopic *zip* transcripts in the head. (F) Elevation of Egfr signaling in the AS through expression of *Egfr-EGFP* causes down-regulation of *zip* expression in DME cells. Scale bar: 50 µm.

An important target of Dpp regulation during DC is *zip*, and we evaluated *zip* expression in *Egfr* mutant embryos and embryos with tissue-specific attenuation of Egfr signaling. *zip* shows two major events of transcriptional upregulation that are relevant to the morphogenetic events during DC: first, a burst of expression occurs in the AS during germband retraction and terminates around the beginning of DC; and, second, upregulation occurs in the DME cells beginning during the germband retraction stage and persists throughout DC [12], [24], [26](Fig. 4A). *Egfr* mutant embryos showed excessive accumulation of *zip* transcripts in the DME cells, in addition to some ectopic *zip* transcription in the epidermis (Fig. 4B). We occasionally found less severely disrupted *Egfr* mutant embryos where the AS was intact; in these *zip* transcripts persisted in the AS during DC, in contrast to wild-type embryos where the AS was devoid of transcripts by this stage (compare Fig. 4C with Fig. 4A). Reduction of Egfr function in the epidermis through expression of *UAS-Egfr^DN^* using the *LE-* and *69B-Gal4* drivers also caused excessive epidermal *zip* expression (data not shown). We previously demonstrated that the tyrosine kinase Ack, a putative negative regulator of Egfr, controls *zip* levels in the AS [26]; we, therefore, also tested the effect of disrupting Egfr specifically in this tissue by expressing *UAS-Egfr^DN^* using the AS-specific *Gal4^c381^* driver. In *Gal4^c381^*>*UAS-Egfr^DN^* embryos we observed ectopic *zip* expression throughout the AS during DC as well as elevated *zip* levels in the head, the latter indicating some degree of cell non-autonomous control of *zip* by Egfr (Fig. 4D).

A major downstream effector for Egfr is Ras and expression of a dominant negative Ras transgene, *RasN17* [92], in the AS was found to be associated with a similar increase in *zip* transcript levels (Fig. 4E). Given the robust levels of *zip* transcripts normally seen in the DME cells, we found it difficult to ascertain if knock down of Egfr in the AS affected *zip* expression in the DME cells, but we suspect that it caused a modest elevation (Fig. 4D). To determine if excessive Egfr signaling in the AS would have the opposite effect on *zip* expression, we expressed the *Egfr-EGFP* transgene with *Gal4^c381^* (Fig. 4F). *zip* levels in the DME cells were decreased in association with excessive Egfr signaling in the AS and we conclude that Egfr represses *zip* transcription in a cell non-autonomous manner during DC.

### Egfr Inhibits Apoptosis in the AS

**Figure 5:**
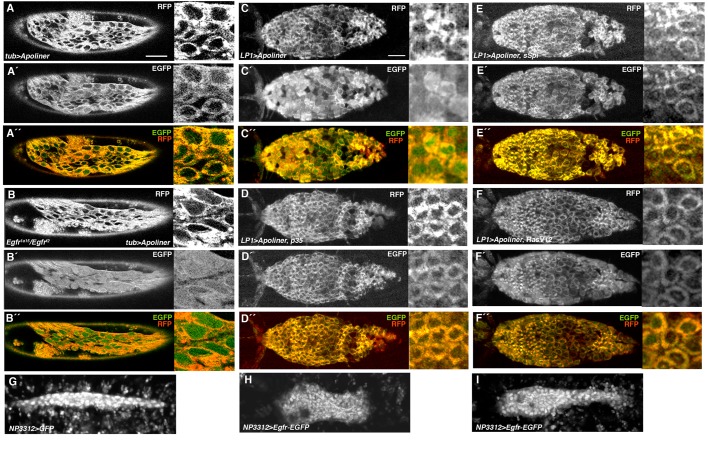
Egfr inhibits apoptosis and morphogenesis in the AS. (A–F´ ´) Apoliner signals in the AS. Apoliner reporter had been expressed either globally with the *tub-Gal4* driver or in the AS using the *LP1-Gal4* driver. For each embryo RFP, EGFP signals and merge are shown. On the right side of each panel is a higher power view of AS cells. In the absence of caspase activity, RFP and EGFP co-localize at various membranes and there is little EGFP signal in the nucleus. In the presence of caspase activity, EGFP is cleaved away from RFP and moves into the nucleus. (A–A´ ´) AS of wild-type embryo prior to germband retraction showing co-localization of RFP and EGFP signals and weak EGFP signals in the nucleus. (B–B´ ´) AS of *Egfr* mutant embryo prior to germband retraction showing strong EGFP signals in the nucleus. (C–C´ ´) AS of wild-type embryo during DC showing strong EGFP signals in the nucleus. (D–D´ ´) AS of p35-expressing embryo during DC showing weak EGFP signals in the nucleus. (E–E´ ´) AS of sSpi-expressing-expressing embryo during DC showing weak EGFP signals in the nucleus. (F–F´ ´) AS of RasV12-expressing embryo during DC showing weak EGFP signals in the nucleus. (G) Still from Movie S7 showing AS of stage 15 wild-type embryo in which GFP had been expressed with the *Gal4^NP3312^* AS driver, showing narrow, tube-like AS. (H, I) Stills from Movies S8 (H) and S9 (I) showing AS of stage 15 embryos in which *Egfr-EGFP* and *GFP-NLS* had been expressed with the double driver combination *Gal4^NP3312^*+ *Gal4^NP5328^* showing failure of AS morphogenesis. The AS in panel H has failed to narrow throughout while that in panel I has failed to narrow at the anterior end. Scale bars: 50 µm (A–B´ ´); 10 µm (C–I).

The similarity of *Egfr* mutant embryonic phenotypes to those of the U-shaped group of genes, which are required for maintenance of the AS, has been noted and studies on fixed preparations suggest premature apoptosis in *Egfr* mutant embryos [35], [93], [94]. Apoptosis of the AS cells contributes to the forces driving DC, and negative regulation of cell death in the AS could be an important component of the participation of Egfr in DC [2], [3]. A negative regulatory role for the Egfr pathway is further supported by the observation that expression of a constitutively active version of Ras, RasV12, in the AS causes the tissue to persist longer than wild-type [95]. Furthermore, Ras appears to negatively regulate apoptosis throughout the embryo, as revealed by acridine orange staining of embryos with global gains or losses of Ras signaling [96]. As described above, AS cells are lost prematurely in *Egfr* mutant embryos (Fig. 2D, F) suggesting an early onset of apoptosis. To visualize the effects of losses or gains of Egfr signaling on AS apoptosis in live embryos, we used the caspase sensor Apoliner [97], [98]. Apoliner consists of a monomeric red fluorescent protein (RFP) tethered to EGFP by a caspase-sensitive linker [97]. Furthermore, the design of the Apoliner construct includes a transmembrane domain that precedes the RFP component while the EGFP component includes a nuclear localization signal (NLS). As a consequence, the two fluorophores co-localize to membranes in live cells lacking caspase activity, but caspase activation in live cells results in separation of the fluorophores, with Apoliner-RFP remaining at membranes while Apoliner-EGFP is translocated to the nucleus due to its NLS. At the beginning of germband retraction in wild-type embryos there was little nuclear EGFP in the AS, indicating minimal caspase activity (Fig. 5 A–A´ ´), but there was strong nuclear EGFP in the AS of *Egfr* mutant embryos at the same stage (Fig. 5 B–B´ ´). As DC proceeded nuclear EGFP accumulated in the AS of wild-type embryos (Fig. 5C–C ´´) and this accumulation could be blocked by expression of the baculovirus caspase inhibitor p35 [99] (Fig. 5D–D ´´). To promote Egfr signaling in the AS, we expressed either sSpi or RasV12 and found that, in both cases, cells showed little nuclear EGFP even late in DC, similar to what was seen with p35 expression (Fig. 5E–F ´´). We conclude that Egfr signaling inhibits caspase activation in the AS. If Egfr impedes apoptosis in the AS, then excessive Egfr signaling might be expected to affect AS morphogenesis. A robust increase in Egfr levels in the AS through expression of *Egfr-EGFP* using the double driver combination *Gal4^NP3312^*+ *GAL4^NP5328^* resulted in a failure of the AS to properly complete morphogenesis (compare Fig. 5G to Fig. 5H, I and Movie S7 to Movies S8 and S9). In addition, the AS of these embryos persisted beyond the normal time of AS programmed cell death.

### Evidence that Ack and Endocytosis Negatively Regulate Egfr Levels in the AS

**Figure 6:**
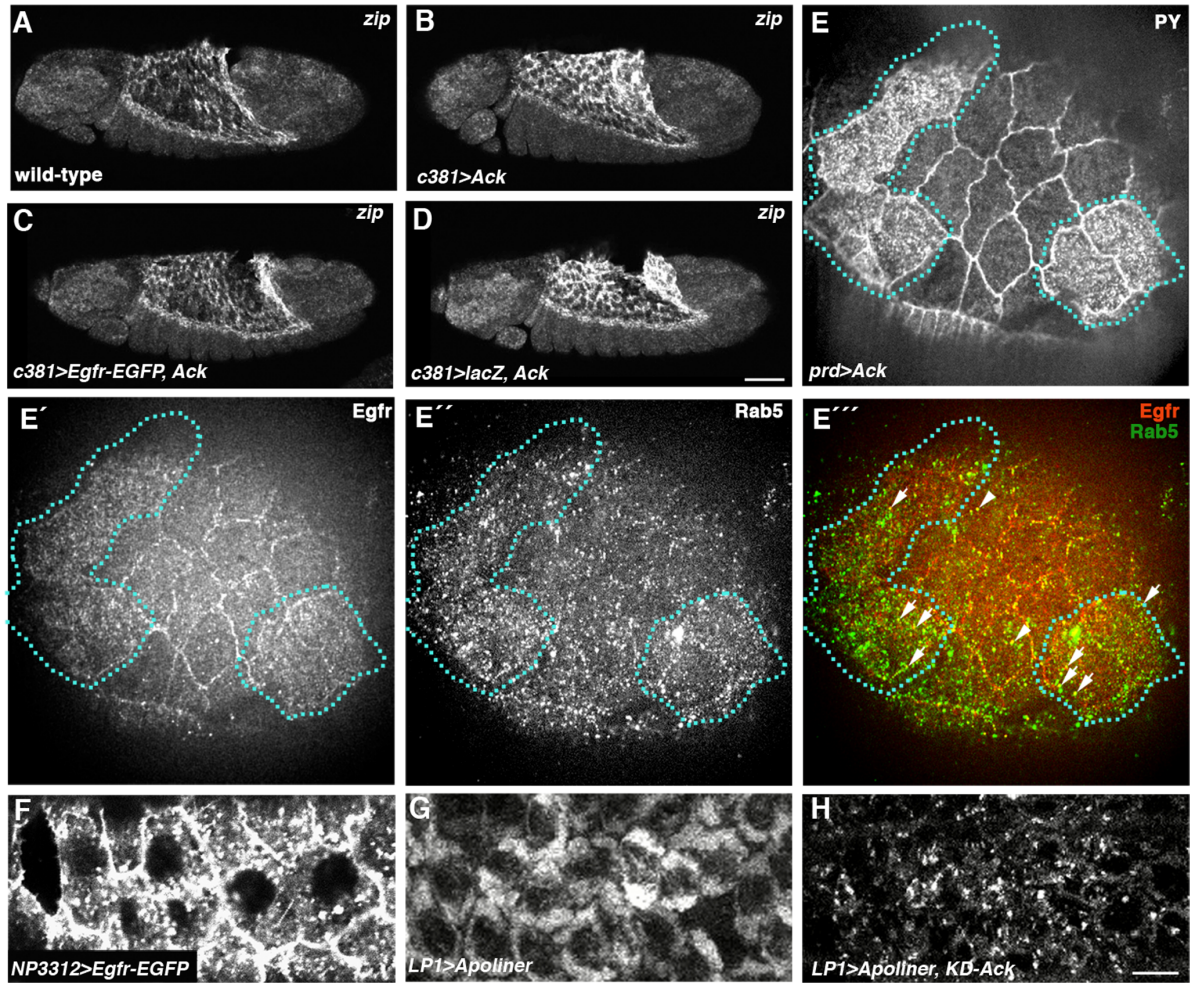
Evidence that Egfr signaling is negatively regulated by endocytosis in the AS. (A–D) *zip* FISH on embryos late in germband retraction. (A) Wild-type embryo showing *zip* expression in AS. (B) Expression of Ack in the AS using the *Gal4^c381^* driver causes an increase in *zip* levels in this tissue relative to wild-type. (C) Ack fails to elevate *zip* levels when co-expressed with Egfr–EGFP. (D) *zip* levels are elevated when Ack is co-expressed with control *lacZ* gene. (E–É ´´) AS in which Ack had been over-expressed in *prd* stripes, triple-stained with anti-phosphotyrosine (anti-PY) (E), anti-Egfr (É) and anti-Rab5 (É ´). (E) Cells over-expressing Ack are marked by high levels of anti-PY (outlined with dotted lines). (É) Egfr shows strong cortical localization in wild-type AS cells but a more cytoplasmic distribution in Ack-over-expressing cells. (É ´) There is an increase in Rab5-positive early endosomes in Ack-over-expressing cells. (É ´´) Merge of panels É and É ´. Arrowheads and arrows mark Egfr-positive early endosomes in wild-type cells and Ack-over-expressing cells, respectively. (F) *Egfr-EGFP* expressed in the AS using the *Gal4^NP3312^* driver shows vesicular accumulation in addition to being at the plasma membrane. (G) AS cells in embryo in which Apoliner has been expressed with *LP1-Gal4* driver showing localization of Apoliner-RFP signal to membranes. (H) AS cells in embryo in which Apoliner and kinase-dead Ack have been co-expressed with *LP1-Gal4* driver showing punctate localization of Apoliner-RFP signal. Scale bars: 50 µm in A-D; 5 µm in E-H.

An important route through which Egfr signaling is down regulated is by clathrin-mediated endocytosis (reviewed in [100]). When imaging Egfr-EGFP in the AS for the apoptosis study, we noticed that in addition to localizing cortically in AS cells, much of the protein appeared to be accumulating in vesicles (Fig. 6F). Given the literature demonstrating that Ack family tyrosine kinases promote down regulation of Egfr by endocytosis and subsequent degradation [29]–[32], we looked for evidence that AS Ack was controlling *zip* expression through down regulation of Egfr in this tissue. Over-expression of Ack in the AS during germband retraction causes a dramatic increase in *zip* levels in this tissue [26](Compare Fig. 6A to Fig. 6B), but co-expression with Egfr-EGFP (but not a control *lacZ* transgene) restored wild-type *zip* levels, suggesting that Ack controls *zip* through down regulation of Egfr (Fig. 6C, D). We subsequently over-expressed Ack in *prd* stripes in the AS and examined the effect on Egfr distribution by comparison with adjacent amnioserosa cells not over-expressing Ack. AS cells with endogenous levels of Ack showed strong cortical Egfr immunostaining as well as staining in cytoplasmic puncta, some of which were Rab5 positive and therefore early endosomes (Fig. 6E–E ´´´). In Ack-over-expressing cells (identified by increased levels of phosphotyrosine [101]) there was a decrease in cortical Egfr staining and an increase in Egfr-positive cytoplasmic puncta, with some of these being Rab5-positive; these cells also showed a general increase in the levels of early endosomes (Fig. 6E–E ´´´). Many of the Egfr-positive puncta in these cells were Rab5-negative and we suspect that they may be multivesicular bodies, where endocytosed Egfr is known to accumulate (reviewed in [100]). The increase in early endosomes in Ack-over-expressing cells indicates that Ack over-expression leads to a general increase in endocytosis in AS cells. In support of this is an observation we made when trying to observe the effects of Ack on apoptosis using the Apoliner reporter. Here, with the expression of kinase-dead Ack, which is more effective than wild-type Ack at inducing *zip* expression [26], a highly punctate distribution of membrane-localized Apoliner-RFP signal was observed while control embryos at the same stage of DC displayed a typical homogeneous distribution (Fig. 6G, H). We interpret this difference as reflecting a general increase in intracellular vesicular traffic, consistent with the effect of Ack over-expression in promoting Egfr endocytosis.

## Discussion

**Figure 7:**
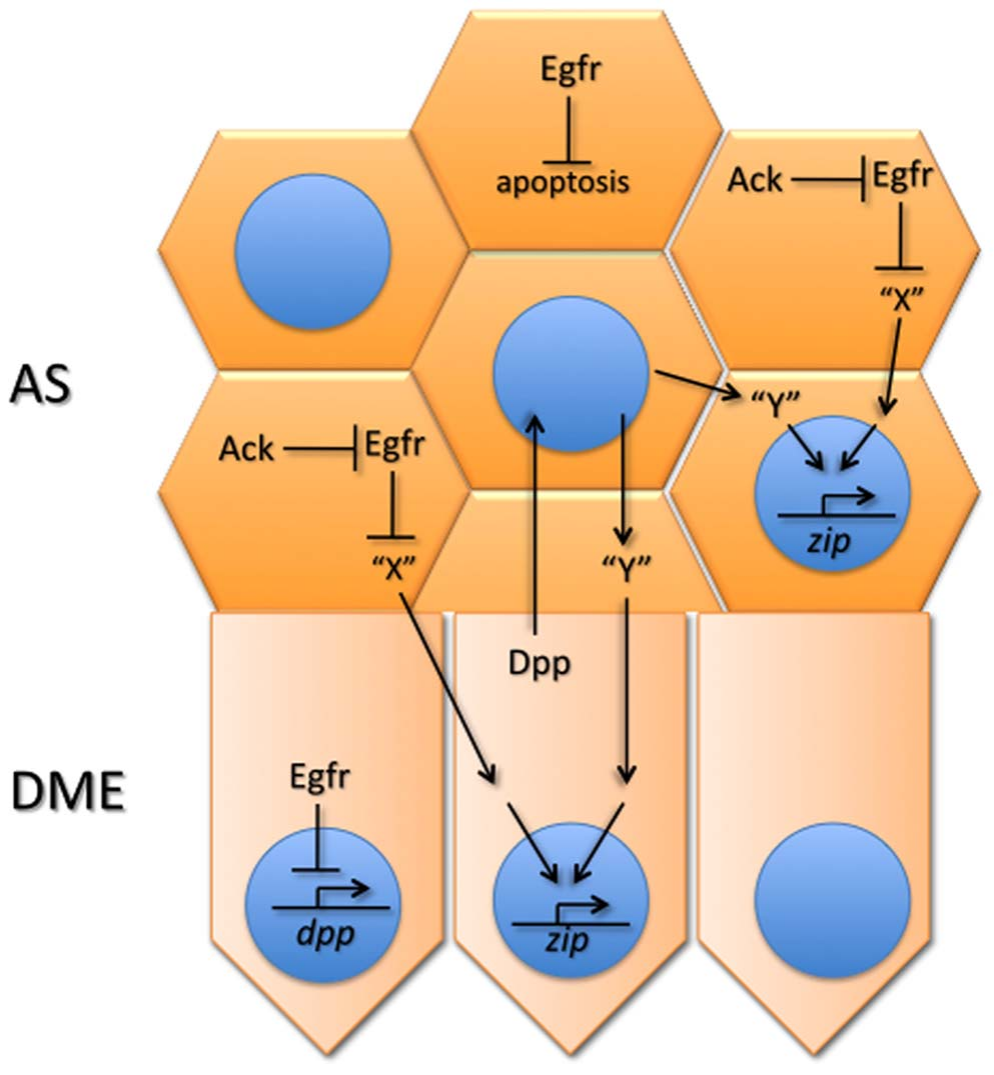
Model for Egfr acting as a brake on DC. Egfr negatively regulates the production and/or secretion of a diffusible signal “X” in the AS (AS) and is itself negatively regulated by Ack through endocytosis. “X” signals into both the AS and the DME cells where it activates a pathway promoting transcription of myosin from the *zip* locus. Previous work from our group and others, and unpublished results from our group, suggest that Dpp from the DME cells diffuses to the AS where it regulates production of a second diffusible signal “Y” providing a parallel input into *zip* transcription. Myosin produced through the cooperation of the two pathways then drives morphogenesis of the AS and DME cells. Egfr additionally regulates this signaling network by negatively regulating *dpp* transcription in the epidermis, including the DME cells. Egfr further regulates AS morphogenesis by inhibiting apoptosis in this tissue.

We have determined that Egfr is required in both the AS and epidermis for DC to proceed normally and our results suggest that Egfr signaling has a least three distinct roles in DC, all of which act to repress morphogenesis (see model in Fig. 7). Egfr is a negative regulator of *dpp* expression in the epidermis as loss of Egfr function in either *Egfr* mutant embryos or as a result of *Egfr^DN^* expression leads to ectopic *dpp* expression. *dpp* is expressed in two stripes during DC, one composed of the DME cells and the other running along the ventrolateral epidermis, where *dpp* expression in the DME cells, but not the ventrolateral stripe, is dependent on a JNK MAPK cascade [13]–[18]. Consistent with the notion that Egfr functions as a negative regulator of *dpp* expression, activation of the Egfr pathway can repress *dpp* expression in either stripe. The down regulation of *dpp* expression in both stripes, however, supports the view that Egfr does not reduce *dpp* transcription by impacting the JNK pathway, in which case we would expect to observe down regulation of *dpp* expression only in the DME stripe. How might Egfr signaling be regulating *dpp* expression? Wingless (Wg) is a diffusible signal required for proper *dpp* expression in both the dorsal and ventrolateral stripes during DC [102], [103]. Egfr negatively regulates Wg levels in the eye imaginal disc by transcriptionally regulating *phyllopod* [104]; we looked for evidence that Egfr might be controlling *dpp* expression through Wg, but altering Egfr signaling had no discernable effect on *phyllopod* transcription or Wg distribution in the embryo (X. C., unpublished results).

The defects in morphogenesis seen in embryos with impaired Egfr signaling are likely at least in part due to misregulated actomyosin contractility. A recurring theme associated with various circumstances of Egfr impairment is the bowed embryo phenotype, where segments are bunched together at the leading edge of the epidermis during DC. We suggest this is due to uneven actomyosin contractility in the dorsal epidermis associated with excessive *zip* expression. The loss of epithelial integrity or “pulling apart” of the head seen in live imaging of Egfr mutant embryos may similarly be due to misregulated actomyosin contractility and/or excessive apoptosis. Previous work has indicated that a major function for Egfr in the head is inhibition of apoptosis, similar to its role in the AS [74], [105], [106]. Parallels between the regulation of head involution and DC have been noted and Egfr may function to modulate these two morphogenetic events through similar mechanisms [107].

While it is likely that increased levels of Dpp in *Egfr* mutant embryos contribute to the elevated *zip* levels, our results of manipulating Egfr signaling support the interpretation of a separate route for *zip* regulation that involves signaling from the AS to both the AS and the epidermis. This signaling is not operating through the regulation of *dpp* expression as impairment of Egfr signaling in the AS does not affect Dpp levels. Thus, we consider this *zip* regulation a second distinct role for Egfr in DC and we believe this signaling is the same as that regulated by Ack in its control of *zip* expression. Consistent with this, gains or losses of Ack do not affect the Dpp pathway, supporting the view that Ack operates in parallel to Dpp signaling [26], [101]. The Ack/Egfr-regulated signal could be a diffusible ligand (“X” in Fig. 7) produced in the AS cells that activates a pathway in the AS and DME cells, thereby driving *zip* expression [26]. Alternatively, Egfr could promote the production of a signal that negatively regulates the pathway required for *zip* expression. Moreover, the upregulation of *zip* expression in Egfr signaling deficient embryos does not appear to be due to premature apoptosis of the AS as elevated *zip* can be seen in Egfr signaling deficient embryos that still have an intact AS. It is possible, however, that the signaling events regulating *zip* expression occur at a stage prior to the stage at which the AS is lost in *Egfr* mutants, i.e. before initiation of germband retraction. Preliminary data leads us to propose that Dpp acts in parallel to produce a second diffusible ligand (“Y” in Fig. 7) that activates a second pathway contributing to *zip* expression (W. S and X. C., unpublished observations). Thus, the interplay between Egfr and Dpp during DC is complex, involving multiple pathways and bidirectional communication between two tissues, and this complex signaling arrangement may function to ensure the coordinated morphogenesis of the AS and epidermis.

A third major role for Egfr in DC is as a negative regulator of apoptosis in the AS. Enhancement of apoptosis accelerates DC whereas suppression of apoptosis slows it, indicating that apoptosis, similar to actomyosin contractility, provides a force for morphogenesis [3]. Thus, down regulation of Egfr in the AS during DC provides two means to accelerate the process: increased myosin expression and increased cell death. The “tweaking” of Egfr function in the AS could constitute an important regulatory mechanism for controlling the rate of closure. We have provided evidence that endocytosis, promoted by Ack, is a route by which Egfr signaling is controlled in the AS cells. Our results suggest that Ack would have a pro-apoptotic role in the AS through promotion of Egfr endocytosis. This in contrast to the *Drosophila* eye in which Ack has an anti-apoptotic function that is independent of Egfr [108].

A recent study has demonstrated that endocytosis in the AS is required for its correct morphogenesis during DC, but this work focused on the role of endocytosis in removing membrane to promote cell shape change [109]. Our results indicate that another route of action for endocytosis in the AS is in regulation of Egfr signaling. It has been suggested that endocytosis could act as a rheostat in which membrane area is adjusted in response to actomyosin contractility [109]; such a rheostat could also be used to adjust Egfr signaling throughout DC.

Additional avenues for Egfr regulation during DC could be control of ligands binding to Egfr and feedback inhibition [110], [111], but we have yet to address these. In summary, we have identified Egfr signaling as an inhibitor of morphogenesis during DC that acts at several distinct levels. Having a single pathway control multiple aspects of this complex process may simplify feedback regulation, ensuring that morphogenesis occurs in a coordinated fashion. In essence, Egfr signaling acts as a brake that can be applied when required to ensure that closure proceeds smoothly and without loss of epidermal integrity. DC shows striking parallels to the healing of induced wounds in the *Drosophila* embryo, with the two processes using similar cytoskeletal and signaling machineries [112]–[114]. Egfr has recently been shown to be required for healing of induced wounds in the embryo and it will be of interest to determine if it uses similar routes of action in this as we have shown in DC [115].

## Materials and Methods

### Fly STOCKS

*Egfr^2C82^* and *Egfr^1F26^* were gifts from T. Schüpbach, *UAS-Egfr-EGFP* from J. Duffy, *UAS-sSpi* from B. Shilo, *UAS-RasN17* from T. Lee, *LE-Gal4* from S. Noselli, *Ubi-DEcadherin-GFP* from H. Oda, *LP1-Gal4* from G. Morata, *Gal4^NP5328^* and *Gal4^NP3312^* from the Kyoto Drosophila Resource Center and *UAS-Apoliner* and *tub-Apoliner* from P.L. Bardet. *Egfr^1a15^* and *Egfr^H25^* were isolated from a collection of EMS-mutagenized second chromosomes [38]. *cn^1^ Egfr^f2^ bw^1^ sp^1^/CyO* flies and all other stocks were obtained from the Bloomington Drosophila Stock Center. Crosses were performed at 25°C.

### Cuticle Preparations

Cuticles were prepared as described but with the fixation step removed [116]. At least 100 embryos were examined in each experiment.

### Immunohistochemistry and RNA in Situ Hybridization

Fixing and antibody staining of embryos were done as previously described [117], [118]. The following primary antibodies were used: mouse anti-phosphotyrosine (Cell Signaling)(1∶1000), rabbit anti-GFP (1∶500)(Sigma), mouse anti-GFP (1∶500)(Sigma), goat anti-Egfr (Santa Cruz)(1∶5), rabbit anti-phospho-MAPK (Cell Signaling)(1∶50) and rabbit anti-Rab5 (Abcam)(1∶1000). All secondary antibodies were from Vector Laboratories and used at a 1∶200 dilution. In situ mRNA hybridizations using digoxigenin-labeled RNA probes and FISH were performed as described [119], [120]. cDNAs for *in situ* hybridization probes were obtained from the Canadian Drosophila Microarray Centre. Fluorescently-stained embryos were examined on either a Zeiss LSM 410 laser-scanning confocal microscope or a Quorum spinning disk confocal microscope, and digoxigenin-labeled embryos imaged using a Zeiss Axioplan 2 microscope. Images were processed in Adobe Photoshop. The genotypes of all fluorescently-stained embryos were established by tracking balancer chromosomes bearing GFP reporters.

### Live Imaging of Embryos

Chromosomes carrying *Ubi-DEcadherin-GFP* in combination with the alleles *Egfr^f2^*, *Egfr^1a15^*, and *Egfr^H25^* were recovered by meiotic recombination. Similarly, the *tub-Apoliner* insertion, which expresses Apoliner under the control of the tubulin 1 α promoter (described in [97]), was recombined with *Egfr^f2^*. All recombinant chromosomes were maintained over so-called “GFP-balancer” chromosomes obtained from the Bloomington Drosophila Stock Center (either *CyO, P{w[+mC] = GAL4-Kr.C}DC3, P{w[+mC] = UAS-GFP.S65T}DC7* or *CyO, P{w[+mC] = GAL4-twi.G}2.2, P{UAS-2xEGFP}AH2.2*). For live imaging experiments *Egfr* mutant embryos were unambiguously identified as those lacking GFP expression derived from the GFP-balancer. Since the onset of the *Egfr* mutant phenotype preceded the timing of GFP expression associated with either GFP-balancer stock, the selection of embryos for live imaging was random and identification of mutant embryos was achieved subsequent to image acquisition. The early onset of the *Egfr* mutant phenotype also preceded the time at which all available amnioserosa specific GAL4 drivers could induce reporter gene expression, and for this reason our analysis of caspase activity in Egfr mutant embryos required using *tub-Apoliner* carried by the maternal parent.

Embryos were prepared for live imaging using the hanging drop protocol, which eliminates effects of compression on the mounted embryo [121]. Time-lapse confocal microscopy was performed using a 20X Plan Apo VC objective on a Nikon Eclipse 90 i microscope with a Nikon D-eclipse C1 scan head. Images were saved as animated projections using the Nikon EZ-C1 3.70 software and further processed using ImageJ (NIH).

## Supporting Information

Movie S1 **Time-lapse showing restricted expression pattern of** ***LE-Gal4*** **driver, revealed using a** ***UAS-GFP-NLS*** **reporter.** Cell outlines were revealed through expression of a *Ubi-DEcadherin-GFP* transgene.
(MOV)

Movie S2 **Time-lapse showing unfolding of the AS as germband retraction proceeds in a** ***Ubi-DEcadherin-GFP-*** **expressing embryo.**
(MOV)

Movie S3 **Time-lapse showing delayed germband retraction and disintegration of AS in** ***Egfr^f2^*** **mutant embryo expressing** ***Ubi-DEcadherin-GFP*** **.**
(MOV)

Movie S4 **Time-lapse showing delayed germband retraction and disintegration of AS in** ***Egfr^f2^*** **/** ***Egfr^H25^*** **mutant embryo expressing** ***Ubi-DEcadherin-GFP*** **.**
(MOV)

Movie S5 **Time-lapse showing bowing of** ***Egfr^1a15^*** **/** ***Egfr^H25^*** **mutant,** ***Ubi-DEcadherin-GFP-*** **expressing embryo.**
(MOV)

Movie S6 **Time-lapse showing morphogenesis of the AS in a** ***Ubi-DEcadherin-GFP-*** **expressing embryo.**
(MOV)

Movie S7 **Time-lapse showing AS of stage 15 wild-type embryo in which GFP had been expressed with the** ***Gal4^NP3312^*** **AS driver, showing morphogenesis of the AS into a narrow, tube-like structure.**
(MOV)

Movie S8 **Time-lapse showing AS of stage 15 embryo in which** ***Egfr-EGFP*** **and** ***GFP-NLS*** **had been expressed with the double driver combination** ***Gal4^NP3312^*** **+** ***Gal4^NP5328^*** **showing failure of AS morphogenesis.**
(MOV)

Movie S9 **Time-lapse showing AS of stage 15 embryo in which** ***Egfr-EGFP*** **and** ***GFP-NLS*** **had been expressed with the double driver combination** ***Gal4^NP3312^*** **+** ***Gal4^NP5328^*** **showing failure of AS morphogenesis.**
(MOV)

## References

[1] Harden N (2002) Signaling pathways directing the movement and fusion of epithelial sheets: lessons from dorsal closure in *Drosophila* . Differentiation 70: 181–203. 12147138 10.1046/j.1432-0436.2002.700408.x

[2] Reed BH, Wilk R, Schock F, Lipshitz HD (2004) Integrin-dependent apposition of *Drosophila* extraembryonic membranes promotes morphogenesis and prevents anoikis. Curr Biol 14: 372–380. 15028211 10.1016/j.cub.2004.02.029

[3] Toyama Y, Peralta XG, Wells AR, Kiehart DP, Edwards GS (2008) Apoptotic force and tissue dynamics during *Drosophila* embryogenesis. Science 321: 1683–1686. 18802000 10.1126/science.1157052 PMC2757114

[4] Kiehart DP, Galbraith CG, Edwards KA, Rickoll WL, Montague RA (2000) Multiple forces contribute to cell sheet morphogenesis for dorsal closure in *Drosophila* . J Cell Biol 149: 471–490. 10769037 10.1083/jcb.149.2.471 PMC2175161

[5] Heisenberg CP (2009) Dorsal closure in *Drosophila*: cells cannot get out of the tight spot. Bioessays 31: 1284–1287. 19882683 10.1002/bies.200900109

[6] Jacinto A, Wood W, Balayo T, Turmaine M, Martinez-Arias A, et al (2000) Dynamic actin-based epithelial adhesion and cell matching during *Drosophila* dorsal closure. Curr Biol 10: 1420–1426. 11102803 10.1016/s0960-9822(00)00796-x

[7] Jacinto A, Wood W, Woolner S, Hiley C, Turner L, et al (2002) Dynamic analysis of actin cable function during *Drosophila* dorsal closure. Curr Biol 12: 1245–1250. 12176336 10.1016/s0960-9822(02)00955-7

[8] Riesgo-Escovar JR, Hafen E (1997) Common and distinct roles of DFos and DJun during *Drosophila* development. Science 278: 669–672. 9381174 10.1126/science.278.5338.669

[9] Zeitlinger J, Kockel L, Peverali FA, Jackson DB, Mlodzik M, et al (1997) Defective dorsal closure and loss of epidermal *decapentaplegic* expression in *Drosophila fos* mutants. EMBO J 16: 7393–7401. 9405368 10.1093/emboj/16.24.7393 PMC1170339

[10] Wada A, Kato K, Uwo MF, Yonemura S, Hayashi S (2007) Specialized extraembryonic cells connect embryonic and extraembryonic epidermis in response to Dpp during dorsal closure in *Drosophila* . Dev Biol 301: 340–349. 17034783 10.1016/j.ydbio.2006.09.020

[11] Ricos MG, Harden N, Sem KP, Lim L, Chia W (1999) Dcdc42 acts in TGF-β signaling during *Drosophila* morphogenesis: distinct roles for the Drac1/JNK and Dcdc42/TGF-β cascades in cytoskeletal regulation. J Cell Sci 112: 1225–1235. 10085257 10.1242/jcs.112.8.1225

[12] Young PE, Richman AM, Ketchum AS, Kiehart DP (1993) Morphogenesis in *Drosophila* requires nonmuscle myosin heavy chain function. Genes Dev 7: 29–41. 8422986 10.1101/gad.7.1.29

[13] Glise B, Noselli S (1997) Coupling of Jun amino-terminal kinase and Decapentaplegic signaling pathways in *Drosophila* morphogenesis. Genes Dev 11: 1738–1747. 9224722 10.1101/gad.11.13.1738

[14] Jackson PD, Hoffmann FM (1994) Embryonic expression patterns of the *Drosophila decapentaplegic* gene: separate regulatory elements control blastoderm expression and lateral ectodermal expression. Dev Dyn 199: 28–44. 8167377 10.1002/aja.1001990104

[15] St Johnston RD, Gelbart WM (1987) Decapentaplegic transcripts are localized along the dorsal-ventral axis of the *Drosophila* embryo. EMBO J 6: 2785–2791. 3119329 10.1002/j.1460-2075.1987.tb02574.x PMC553704

[16] Sluss HK, Davis RJ (1997) Embryonic morphogenesis signaling pathway mediated by JNK targets the transcription factor JUN and the TGF-β homologue *decapentaplegic* . J Cell Biochem 67: 1–12. 9328834

[17] Riesgo-Escovar JR, Hafen E (1997) *Drosophila* Jun kinase regulates expression of *decapentaplegic* via the ETS-domain protein Aop and the AP-1 transcription factor DJun during dorsal closure. Genes Dev 11: 1717–1727. 9224720 10.1101/gad.11.13.1717

[18] Hou XS, Goldstein ES, Perrimon N (1997) *Drosophila* Jun relays the Jun amino-terminal kinase signal transduction pathway to the Decapentaplegic signal transduction pathway in regulating epithelial cell sheet movement. Genes Dev 11: 1728–1737. 9224721 10.1101/gad.11.13.1728

[19] Childs SR, Wrana JL, Arora K, Attisano L, O'Connor MB, et al (1993) Identification of a *Drosophila* activin receptor. Proc Natl Acad Sci U S A 90: 9475–9479. 8415726 10.1073/pnas.90.20.9475 PMC47591

[20] Affolter M, Nellen D, Nussbaumer U, Basler K (1994) Multiple requirements for the receptor serine/threonine kinase *thick veins* reveal novel functions of TGF β homologs during *Drosophila* embryogenesis. Development 120: 3105–3117. 7720555 10.1242/dev.120.11.3105

[21] Penton A, Chen Y, Staehling-Hampton K, Wrana JL, Attisano L, et al (1994) Identification of two bone morphogenetic protein type I receptors in Drosophila and evidence that Brk25D is a Decapentaplegic receptor. Cell 78: 239–250. 8044838 10.1016/0092-8674(94)90294-1

[22] Letsou A, Arora K, Wrana JL, Simin K, Twombly V, et al (1995) Drosophila Dpp signaling is mediated by the *punt* gene product: a dual ligand-binding type II receptor of the TGF β receptor family. Cell 80: 899–908. 7697720 10.1016/0092-8674(95)90293-7

[23] Ruberte E, Marty T, Nellen D, Affolter M, Basler K (1995) An absolute requirement for both the type II and type I receptors, Punt and Thick veins, for Dpp signaling in vivo. Cell 80: 889–897. 7697719 10.1016/0092-8674(95)90292-9

[24] Arquier N, Perrin L, Manfruelli P, Semeriva M (2001) The *Drosophila* tumor suppressor gene *lethal(2)giant larvae* is required for the emission of the Decapentaplegic signal. Development 128: 2209–2220. 11493541 10.1242/dev.128.12.2209

[25] Fernandez BG, Arias AM, Jacinto A (2007) Dpp signalling orchestrates dorsal closure by regulating cell shape changes both in the amnioserosa and in the epidermis. Mech Dev 124: 884–897. 17950580 10.1016/j.mod.2007.09.002

[26] Zahedi B, Shen W, Xu X, Chen X, Mahey M, et al (2008) Leading edge-secreted Dpp cooperates with ACK-dependent signaling from the amnioserosa to regulate myosin levels during dorsal closure. Dev Dyn 237: 2936–2946. 18816840 10.1002/dvdy.21722

[27] Franke JD, Montague RA, Kiehart DP (2005) Nonmuscle myosin II generates forces that transmit tension and drive contraction in multiple tissues during dorsal closure. Curr Biol 15: 2208–2221. 16360683 10.1016/j.cub.2005.11.064

[28] Hopper NA, Lee J, Sternberg PW (2000) ARK-1 inhibits EGFR signaling in C. elegans. Mol Cell 6: 65–75. 10949028

[29] Lin Q, Lo CG, Cerione RA, Yang W (2002) The Cdc42 target ACK2 interacts with sorting nexin 9 (SH3PX1) to regulate epidermal growth factor receptor degradation. J Biol Chem 277: 10134–10138. Epub 12002 Jan 10117. 10.1074/jbc.M110329200 11799118

[30] Shen F, Lin Q, Gu Y, Childress C, Yang W (2007) Activated Cdc42-associated kinase 1 is a component of EGF receptor signaling complex and regulates EGF receptor degradation. Mol Biol Cell 18: 732–742. 17182860 10.1091/mbc.E06-02-0142 PMC1805115

[31] Lin Q, Wang J, Childress C, Sudol M, Carey DJ, et al (2010) HECT E3 ubiquitin ligase Nedd4–1 ubiquitinates ACK and regulates epidermal growth factor (EGF)-induced degradation of EGF receptor and ACK. Mol Cell Biol 30: 1541–1554. 20086093 10.1128/MCB.00013-10 PMC2832494

[32] Grovdal LM, Johannessen LE, Rodland MS, Madshus IH, Stang E (2008) Dysregulation of Ack1 inhibits down-regulation of the EGF receptor. Exp Cell Res 314: 1292–1300. 18262180 10.1016/j.yexcr.2007.12.017

[33] Schweitzer R, Shilo BZ (1997) A thousand and one roles for the *Drosophila* EGF receptor. Trends Genet 13: 191–196. 9154002 10.1016/s0168-9525(97)01091-3

[34] Shilo BZ (2003) Signaling by the *Drosophila* epidermal growth factor receptor pathway during development. Exp Cell Res 284: 140–149. 12648473 10.1016/s0014-4827(02)00094-0

[35] Clifford R, Schupbach T (1992) The torpedo (DER) receptor tyrosine kinase is required at multiple times during *Drosophila* embryogenesis. Development 115: 853–872. 1425358 10.1242/dev.115.3.853

[36] Clifford RJ, Schupbach T (1989) Coordinately and differentially mutable activities of *torpedo*, the *Drosophila* melanogaster homolog of the vertebrate EGF receptor gene. Genetics 123: 771–787. 2515109 10.1093/genetics/123.4.771 PMC1203888

[37] Nusslein-Volhard C, Wieschaus E, Kluding H (1984) Mutations affecting the pattern of the larval cuticle in *Drosophila melanogaster* I. Zygotic loci on the second chromosome. Roux's Arch Dev Biol 193: 267–282. 10.1007/BF00848156 28305337

[38] Royzman I, Whittaker AJ, Orr-Weaver TL (1997) Mutations in *Drosophila* DP and E2F distinguish G1-S progression from an associated transcriptional program. Genes Dev 11: 1999–2011. 9271122 10.1101/gad.11.15.1999 PMC316409

[39] Perkins LA, Johnson MR, Melnick MB, Perrimon N (1996) The nonreceptor protein tyrosine phosphatase corkscrew functions in multiple receptor tyrosine kinase pathways in *Drosophila* . Dev Biol 180: 63–81. 8948575 10.1006/dbio.1996.0285

[40] Brand AH, Perrimon N (1993) Targeted gene expression as a means of altering cell fates and generating dominant phenotypes. Development 118: 401–415. 8223268 10.1242/dev.118.2.401

[41] Hinz U, Giebel B, Campos-Ortega JA (1994) The basic-helix-loop-helix domain of Drosophila lethal of scute protein is sufficient for proneural function and activates neurogenic genes. Cell 76: 77–87. 8287481 10.1016/0092-8674(94)90174-0

[42] Manseau L, Baradaran A, Brower D, Budhu A, Elefant F, et al (1997) GAL4 enhancer traps expressed in the embryo, larval brain, imaginal discs, and ovary of *Drosophila* . Dev Dyn 209: 310–322. 9215645 10.1002/(SICI)1097-0177(199707)209:3<310::AID-AJA6>3.0.CO;2-L

[43] Gabay L, Seger R, Shilo BZ (1997) MAP kinase in situ activation atlas during *Drosophila* embryogenesis. Development 124: 3535–3541. 9342046 10.1242/dev.124.18.3535

[44] Gabay L, Seger R, Shilo BZ (1997) In situ activation pattern of *Drosophila* EGF receptor pathway during development. Science 277: 1103–1106. 9262480 10.1126/science.277.5329.1103

[45] Oda H, Tsukita S (2001) Real-time imaging of cell-cell adherens junctions reveals that *Drosophila* mesoderm invagination begins with two phases of apical constriction of cells. J Cell Sci 114: 493–501. 11171319 10.1242/jcs.114.3.493

[46] Byars CL, Bates KL, Letsou A (1999) The dorsal-open group gene *raw* is required for restricted DJNK signaling during closure. Development 126: 4913–4923. 10518507 10.1242/dev.126.21.4913

[47] Wei HC, Sanny J, Shu H, Baillie DL, Brill JA, et al (2003) The Sac1 lipid phosphatase regulates cell shape change and the JNK cascade during dorsal closure in *Drosophila* . Curr Biol 13: 1882–1887. 14588244 10.1016/j.cub.2003.09.056

[48] Martin-Blanco E, Gampel A, Ring J, Virdee K, Kirov N, et al (1998) *puckered* encodes a phosphatase that mediates a feedback loop regulating JNK activity during dorsal closure in *Drosophila* . Genes Dev 12: 557–570. 9472024 10.1101/gad.12.4.557 PMC316530

[49] Yagi Y, Hayashi S (1997) Role of the *Drosophila* EGF receptor in determination of the dorsoventral domains of escargot expression during primary neurogenesis. Genes Cells 2: 41–53. 9112439 10.1046/j.1365-2443.1997.d01-282.x

[50] Chen CK, Kuhnlein RP, Eulenberg KG, Vincent S, Affolter M, et al (1998) The transcription factors KNIRPS and KNIRPS RELATED control cell migration and branch morphogenesis during *Drosophila* tracheal development. Development 125: 4959–4968. 9811580 10.1242/dev.125.24.4959

[51] Dobens LL, Peterson JS, Treisman J, Raftery LA (2000) *Drosophila bunched* integrates opposing DPP and EGF signals to set the operculum boundary. Development 127: 745–754. 10648233 10.1242/dev.127.4.745

[52] Kumar JP, Moses K (2001) The EGF receptor and notch signaling pathways control the initiation of the morphogenetic furrow during *Drosophila* eye development. Development 128: 2689–2697. 11526075 10.1242/dev.128.14.2689

[53] Chen Y, Schupbach T (2006) The role of brinker in eggshell patterning. Mech Dev 123: 395–406. 16707253 10.1016/j.mod.2006.03.007

[54] Carneiro K, Fontenele M, Negreiros E, Lopes E, Bier E, et al (2006) Graded maternal short gastrulation protein contributes to embryonic dorsal-ventral patterning by delayed induction. Dev Biol 296: 203–218. 16781701 10.1016/j.ydbio.2006.04.453

[55] Shravage BV, Altmann G, Technau M, Roth S (2007) The role of Dpp and its inhibitors during eggshell patterning in *Drosophila* . Development 134: 2261–2271. 17507396 10.1242/dev.02856

[56] Yan SJ, Zartman JJ, Zhang M, Scott A, Shvartsman SY, et al (2009) Bistability coordinates activation of the EGFR and DPP pathways in *Drosophila* vein differentiation. Mol Syst Biol 5: 278. 19536201 10.1038/msb.2009.35 PMC2710866

[57] Liu M, Lim TM, Cai Y (2010) The *Drosophila* female germline stem cell lineage acts to spatially restrict DPP function within the niche. Sci Signal 3: ra57. 20664066 10.1126/scisignal.2000740

[58] Szuts D, Bienz M (2000) An autoregulatory function of Dfos during *Drosophila* endoderm induction. Mech Dev 98: 71–76. 11044608 10.1016/s0925-4773(00)00455-x

[59] Kopp A, Blackman RK, Duncan I (1999) Wingless, decapentaplegic and EGF receptor signaling pathways interact to specify dorso-ventral pattern in the adult abdomen of *Drosophila* . Development 126: 3495–3507. 10409497 10.1242/dev.126.16.3495

[60] Wappner P, Gabay L, Shilo BZ (1997) Interactions between the EGF receptor and DPP pathways establish distinct cell fates in the tracheal placodes. Development 124: 4707–4716. 9409686 10.1242/dev.124.22.4707

[61] Peri F, Roth S (2000) Combined activities of Gurken and decapentaplegic specify dorsal chorion structures of the *Drosophila* egg. Development 127: 841–850. 10648242 10.1242/dev.127.4.841

[62] Deng WM, Bownes M (1997) Two signalling pathways specify localised expression of the Broad-Complex in *Drosophila* eggshell patterning and morphogenesis. Development 124: 4639–4647. 9409680 10.1242/dev.124.22.4639

[63] de Celis JF (1997) Expression and function of decapentaplegic and thick veins during the differentiation of the veins in the *Drosophila* wing. Development 124: 1007–1018. 9056776 10.1242/dev.124.5.1007

[64] Carmena A, Gisselbrecht S, Harrison J, Jimenez F, Michelson AM (1998) Combinatorial signaling codes for the progressive determination of cell fates in the *Drosophila* embryonic mesoderm. Genes Dev 12: 3910–3922. 9869644 10.1101/gad.12.24.3910 PMC317272

[65] Martin-Blanco E, Roch F, Noll E, Baonza A, Duffy JB, et al (1999) A temporal switch in DER signaling controls the specification and differentiation of veins and interveins in the *Drosophila* wing. Development 126: 5739–5747. 10572049 10.1242/dev.126.24.5739

[66] Jordan KC, Clegg NJ, Blasi JA, Morimoto AM, Sen J, et al (2000) The homeobox gene *mirror* links EGF signalling to embryonic dorso-ventral axis formation through notch activation. Nat Genet 24: 429–433. 10742112 10.1038/74294

[67] Dequier E, Souid S, Pal M, Maroy P, Lepesant JA, et al (2001) Top-DER- and Dpp-dependent requirements for the *Drosophila fos/kayak* gene in follicular epithelium morphogenesis. Mech Dev 106: 47–60. 11472834 10.1016/s0925-4773(01)00418-x

[68] Shirai T, Maehara A, Kiritooshi N, Matsuzaki F, Handa H, et al (2003) Differential requirement of EGFR signaling for the expression of defective proventriculus gene in the *Drosophila* endoderm and ectoderm. Biochem Biophys Res Commun 311: 473–477. 14592438 10.1016/j.bbrc.2003.10.017

[69] Motola S, Neuman-Silberberg FS (2004) *spoonbill*, a new *Drosophila* female-sterile mutation, interferes with chromosome organization and dorsal-ventral patterning of the egg. Dev Dyn 230: 535–545. 15188438 10.1002/dvdy.20066

[70] de Navascues J, Modolell J (2007) *tailup*, a LIM-HD gene, and Iro-C cooperate in *Drosophila* dorsal mesothorax specification. Development 134: 1779–1788. 17409113 10.1242/dev.02844

[71] O'Keefe DD, Prober DA, Moyle PS, Rickoll WL, Edgar BA (2007) Egfr/Ras signaling regulates DE-cadherin/Shotgun localization to control vein morphogenesis in the *Drosophila* wing. Dev Biol 311: 25–39. 17888420 10.1016/j.ydbio.2007.08.003 PMC2128780

[72] Yakoby N, Lembong J, Schupbach T, Shvartsman SY (2008) *Drosophila* eggshell is patterned by sequential action of feedforward and feedback loops. Development 135: 343–351. 18077592 10.1242/dev.008920

[73] Kim SY, Jung KI, Kim SH, Jeon SH (2008) Dpp represses eagle expression at short-range, but can repress its expression at a long-range via EGFR signal repression. Mol Cells 26: 576–582. 18779662

[74] Chang T, Shy D, Hartenstein V (2003) Antagonistic relationship between Dpp and EGFR signaling in *Drosophila* head patterning. Dev Biol 263: 103–113. 14568549 10.1016/s0012-1606(03)00448-2

[75] Crozatier M, Glise B, Vincent A (2002) Connecting Hh, Dpp and EGF signalling in patterning of the *Drosophila* wing; the pivotal role of collier/knot in the AP organiser. Development 129: 4261–4269. 12183378 10.1242/dev.129.18.4261

[76] Kubota K, Goto S, Eto K, Hayashi S (2000) EGF receptor attenuates Dpp signaling and helps to distinguish the wing and leg cell fates in *Drosophila* . Development 127: 3769–3776. 10934021 10.1242/dev.127.17.3769

[77] Letizia A, Barrio R, Campuzano S (2007) Antagonistic and cooperative actions of the EGFR and Dpp pathways on the iroquois genes regulate *Drosophila* mesothorax specification and patterning. Development 134: 1337–1346. 17329358 10.1242/dev.02823

[78] Sotillos S, De Celis JF (2005) Interactions between the Notch, EGFR, and decapentaplegic signaling pathways regulate vein differentiation during *Drosophila* pupal wing development. Dev Dyn 232: 738–752. 15704120 10.1002/dvdy.20270

[79] Szuts D, Eresh S, Bienz M (1998) Functional intertwining of Dpp and EGFR signaling during *Drosophila* endoderm induction. Genes Dev 12: 2022–2035. 9649506 10.1101/gad.12.13.2022 PMC316971

[80] von Ohlen T, Doe CQ (2000) Convergence of Dorsal, Dpp, and Egfr signaling pathways subdivides the *Drosophila* neuroectoderm into three dorsal-ventral columns. Dev Biol 224: 362–372. 10926773 10.1006/dbio.2000.9789

[81] Wahlstrom G, Norokorpi HL, Heino TI (2006) *Drosophila* alpha-actinin in ovarian follicle cells is regulated by EGFR and Dpp signalling and required for cytoskeletal remodelling. Mech Dev 123: 801–818. 17008069 10.1016/j.mod.2006.08.004

[82] Kretzschmar M, Doody J, Massague J (1997) Opposing BMP and EGF signalling pathways converge on the TGF-β family mediator Smad1. Nature 389: 618–622. 9335504 10.1038/39348

[83] Radke K, Johnson K, Guo R, Davidson A, Ambrosio L (2001) *Drosophila*-raf acts to elaborate dorsoventral pattern in the ectoderm of developing embryos. Genetics 159: 1031–1044. 11729151 10.1093/genetics/159.3.1031 PMC1461885

[84] Lamka ML, Lipshitz HD (1999) Role of the amnioserosa in germ band retraction of the *Drosophila melanogaster* embryo. Dev Biol 214: 102–112. 10491260 10.1006/dbio.1999.9409

[85] Reed BH, Wilk R, Lipshitz HD (2001) Downregulation of Jun kinase signaling in the amnioserosa is essential for dorsal closure of the *Drosophila* embryo. Curr Biol 11: 1098–1108. 11509232 10.1016/s0960-9822(01)00318-9

[86] Stronach BE, Perrimon N (2001) Investigation of leading edge formation at the interface of amnioserosa and dorsal ectoderm in the *Drosophila* embryo. Development 128: 2905–2913. 11532914 10.1242/dev.128.15.2905

[87] Conder R, Yu H, Ricos M, Hing H, Chia W, et al (2004) dPak is required for integrity of the leading edge cytoskeleton during *Drosophila* dorsal closure but does not signal through the JNK cascade. Dev Biol 276: 378–390. 15581872 10.1016/j.ydbio.2004.08.044

[88] Scuderi A, Letsou A (2005) Amnioserosa is required for dorsal closure in *Drosophila* . Dev Dyn 232: 791–800. 15704109 10.1002/dvdy.20306

[89] Lada K, Gorfinkiel N, Martinez Arias A (2012) Interactions between the amnioserosa and the epidermis revealed by the function of the *u-shaped* gene. Biol Open 1: 353–361. 23213425 10.1242/bio.2012497 PMC3509461

[90] Schweitzer R, Shaharabany M, Seger R, Shilo BZ (1995) Secreted Spitz triggers the DER signaling pathway and is a limiting component in embryonic ventral ectoderm determination. Genes Dev 9: 1518–1529. 7601354 10.1101/gad.9.12.1518

[91] Goentoro LA, Reeves GT, Kowal CP, Martinelli L, Schupbach T, et al (2006) Quantifying the Gurken morphogen gradient in Drosophila oogenesis. Dev Cell 11: 263–272. 16890165 10.1016/j.devcel.2006.07.004 PMC4091837

[92] Lee T, Feig L, Montell DJ (1996) Two distinct roles for Ras in a developmentally regulated cell migration. Development 122: 409–418. 8625792 10.1242/dev.122.2.409

[93] Frank LH, Rushlow C (1996) A group of genes required for maintenance of the amnioserosa tissue in *Drosophila* . Development 122: 1343–1352. 8625823 10.1242/dev.122.5.1343

[94] Goldman-Levi R, Miller C, Greenberg G, Gabai E, Zak NB (1996) Cellular pathways acting along the germband and in the amnioserosa may participate in germband retraction of the *Drosophila melanogaster* embryo. Int J Dev Biol 40: 1043–1051. 8946251

[95] Mohseni N, McMillan SC, Chaudhary R, Mok J, Reed BH (2009) Autophagy promotes caspase-dependent cell death during Drosophila development. Autophagy 5: 329–338. 19066463 10.4161/auto.5.3.7444

[96] Kurada P, White K (1998) Ras promotes cell survival in Drosophila by downregulating hid expression. Cell 95: 319–329. 9814703 10.1016/s0092-8674(00)81764-x

[97] Bardet PL, Kolahgar G, Mynett A, Miguel-Aliaga I, Briscoe J, et al (2008) A fluorescent reporter of caspase activity for live imaging. Proc Natl Acad Sci U S A 105: 13901–13905. 18779587 10.1073/pnas.0806983105 PMC2544551

[98] Cormier O, Mohseni N, Voytyuk I, Reed BH (2012) Autophagy can promote but is not required for epithelial cell extrusion in the amnioserosa of the *Drosophila* embryo. Autophagy 8: 252–264. 22240588 10.4161/auto.8.2.18618 PMC3336078

[99] Clem RJ, Fechheimer M, Miller LK (1991) Prevention of apoptosis by a baculovirus gene during infection of insect cells. Science 254: 1388–1390. 1962198 10.1126/science.1962198

[100] Eden ER, White IJ, Futter CE (2009) Down-regulation of epidermal growth factor receptor signalling within multivesicular bodies. Biochem Soc Trans 37: 173–177. 19143625 10.1042/BST0370173

[101] Sem KP, Zahedi B, Tan I, Deak M, Lim L, et al (2002) ACK family tyrosine kinase activity is a component of Dcdc42 Signaling during dorsal closure in *Drosophila melanogaster* . Mol Cell Biol 22: 3685–3697. 11997505 10.1128/MCB.22.11.3685-3697.2002 PMC133815

[102] Morel V, Arias AM (2004) Armadillo/beta-catenin-dependent Wnt signalling is required for the polarisation of epidermal cells during dorsal closure in *Drosophila* . Development 131: 3273–3283. 15226252 10.1242/dev.01217

[103] McEwen DG, Cox RT, Peifer M (2000) The canonical Wg and JNK signaling cascades collaborate to promote both dorsal closure and ventral patterning. Development 127: 3607–3617. 10903184 10.1242/dev.127.16.3607

[104] Nagaraj R, Banerjee U (2009) Regulation of Notch and Wingless signalling by phyllopod, a transcriptional target of the EGFR pathway. EMBO J 28: 337–346. 19153610 10.1038/emboj.2008.286 PMC2646148

[105] Dumstrei K, Nassif C, Abboud G, Aryai A, Hartenstein V (1998) EGFR signaling is required for the differentiation and maintenance of neural progenitors along the dorsal midline of the *Drosophila* embryonic head. Development 125: 3417–3426. 9693145 10.1242/dev.125.17.3417

[106] Dumstrei K, Wang F, Shy D, Tepass U, Hartenstein V (2002) Interaction between EGFR signaling and DE-cadherin during nervous system morphogenesis. Development 129: 3983–3994. 12163402 10.1242/dev.129.17.3983

[107] VanHook A, Letsou A (2008) Head involution in *Drosophila*: genetic and morphogenetic connections to dorsal closure. Dev Dyn 237: 28–38. 18095344 10.1002/dvdy.21405

[108] Schoenherr JA, Drennan JM, Martinez JS, Chikka MR, Hall MC, et al (2012) *Drosophila* activated Cdc42 kinase has an anti-apoptotic function. PLoS Genet 8: e1002725. 22615583 10.1371/journal.pgen.1002725 PMC3355085

[109] Mateus AM, Gorfinkiel N, Schamberg S, Martinez Arias A (2011) Endocytic and recycling endosomes modulate cell shape changes and tissue behaviour during morphogenesis in *Drosophila* . PLoS One 6: e18729. 21533196 10.1371/journal.pone.0018729 PMC3077405

[110] Freeman M (2000) Feedback control of intercellular signalling in development. Nature 408: 313–319. 11099031 10.1038/35042500

[111] Segatto S, Anastasi S, Alema S (2011) Regulation of epidermal growth factor signalling by inducible feedback inhibitors. J Cell Sci 124: 1785–1793. 21576352 10.1242/jcs.083303

[112] Wood W, Jacinto A, Grose R, Woolner S, Gale J, et al (2002) Wound healing recapitulates morphogenesis in *Drosophila* embryos. Nat Cell Biol 4: 907–912. 12402048 10.1038/ncb875

[113] Martin P, Parkhurst SM (2004) Parallels between tissue repair and embryo morphogenesis. Development 131: 3021–3034. 15197160 10.1242/dev.01253

[114] Campos I, Geiger JA, Santos AC, Carlos V, Jacinto A (2010) Genetic screen in *Drosophila* melanogaster uncovers a novel set of genes required for embryonic epithelial repair. Genetics 184: 129–140. 19884309 10.1534/genetics.109.110288 PMC2815911

[115] Geiger JA, Carvalho L, Campos I, Santos AC, Jacinto A (2011) Hole-in-one mutant phenotypes link EGFR/ERK signaling to epithelial tissue repair in *Drosophila* . PLoS One 6: e28349. 22140578 10.1371/journal.pone.0028349 PMC3226689

[116] Ashburner M (1989) *Drosophila*: A Laboratory Manual. Cold Spring Harbor, NY: Cold Spring Harbor Laboratory Press.

[117] Harden N, Lee J, Loh HY, Ong YM, Tan I, et al (1996) A *Drosophila* homolog of the Rac- and Cdc42-activated serine/threonine kinase PAK is a potential focal adhesion and focal complex protein that colocalizes with dynamic actin structures. Mol Cell Biol 16: 1896–1908. 8628256 10.1128/mcb.16.5.1896 PMC231177

[118] Harden N, Ricos M, Ong YM, Chia W, Lim L (1999) Participation of small GTPases in dorsal closure of the *Drosophila* embryo: distinct roles for Rho subfamily proteins in epithelial morphogenesis. J Cell Sci 112: 273–284. 9885281 10.1242/jcs.112.3.273

[119] Van Vactor D, Kopczynski C (1999) Anatomical Techniques for Analysis of Nervous System Development in the *Drosophila* Embryo. In: Richter J, editor. A Comparative Methods Approach to the Study of Oocytes and Embryos. New York: Oxford University Press. 490–513.

[120] Lecuyer E, Parthasarathy N, Krause HM (2007) Fluorescent *in situ* Hybridization Protocols in *Drosophila* Embryos and Tissues. In: Dahmann C, editor. *Drosophila*: Methods and Protocols. Totowa, NJ: Humana Press. 10.1007/978-1-59745-583-1_18 18641955

[121] Reed BH, McMillan SC, Chaudhary R (2009) The preparation of *Drosophila* embryos for live-imaging using the hanging drop protocol. J Vis Exp. 10.3791/1206 PMC2789762 19287353

